# Genome-Scale Metabolic Modeling of Glioblastoma Reveals Promising Targets for Drug Development

**DOI:** 10.3389/fgene.2020.00381

**Published:** 2020-04-17

**Authors:** Ida Larsson, Mathias Uhlén, Cheng Zhang, Adil Mardinoglu

**Affiliations:** ^1^Department of Immunology, Genetics and Pathology, Uppsala University, Uppsala, Sweden; ^2^Science for Life Laboratory, KTH – Royal Institute of Technology, Stockholm, Sweden; ^3^Centre for Host-Microbiome Interactions, Dental Institute, King’s College London, London, United Kingdom

**Keywords:** glioblastoma, GBM, genome-scale metabolic models, GEMs, systems biology

## Abstract

Glioblastoma (GBM) is an aggressive type of brain cancer with a poor prognosis for affected patients. The current line of treatment only gives the patients a survival time of on average 15 months. In this work, we use genome-scale metabolic models (GEMs) together with other systems biology tools to examine the global transcriptomics-data of GBM-patients obtained from The Cancer Genome Atlas (TCGA). We reveal the molecular mechanisms underlying GBM and identify potential therapeutic targets for effective treatment of patients. The work presented consists of two main parts. The first part stratifies the patients into two groups, high and low survival, and compares their gene expression. The second part uses GBM and healthy brain tissue GEMs to simulate gene knockout in a GBM cell model to find potential therapeutic targets and predict their side effect in healthy brain tissue. We (1) find that genes upregulated in the patients with low survival are linked to various stages of the glioma invasion process, and (2) identify five essential genes for GBM, whose inhibition is non-toxic to healthy brain tissue, therefore promising to investigate further as therapeutic targets.

## Introduction

Glioblastoma (GBM) is an aggressive type of brain cancer. Compared to other tumors originating in the brain or central nervous system (CNS), GBM has a high incidence rate (3.14 in 100,000) and low survival estimate, less than 5% of all patients survived 5 years after diagnosis (Ostrom et al., 2013). Hence, there is an urgent need to reveal the molecular mechanisms underlying GBM and identify potential therapeutic targets for effective treatment of GBM patients.

Accumulating evidence indicate that GBM is a highly heterogeneous disease, both in terms of genetic differences and cell of origin (Alcantara Llaguno and Parada, 2016; Wirsching et al., 2016). However, what seems to be shared among all malignant gliomas is their aggressive and unique invasion pattern, where single tumor cells migrate away from the primary tumor and invade surrounding tissue (Goldbrunner et al., 1999). One consequence of this invasion pattern is the difficulty to surgically remove all tumor cells and the high extent of tumor recurrence (Holland, 2000). In 2000 Hanahan and Weinberg published their first work about the Hallmarks of Cancer, where they specify six features acquired during tumor development that distinguish malignant cancers in general from normal cells. One of these hallmarks concerns invasion and metastasis (Hanahan and Weinberg, 2000). Firstly, malignant cancers have the ability to invade the neighboring tissue. Secondly, they can spread to other organs and form secondary tumors, often taking advantage of the bloodstream or lymphatic system (Strachan et al., 2014). While it is extremely rare to observe metastasis of gliomas outside the brain, they are proficient at intra-organ invasion. Glioma cells interact with their microenvironment and migrate along the pre-existing architecture in the brain, such as blood vessels and nerve tracts, adapting their shape to the structure they are moving along. Hence, invading glioma cells originating from the same tumor can vary greatly in shape (Cuddapah et al., 2014).

Many of the cell’s systems undergo major reprogramming as part of the tumorigenesis of GBM, some of the most significant alterations can be observed in the metabolic system (Agnihotri and Zadeh, 2016; Ozcan and Cakir, 2016). Metabolism is the collective name for the many integrated biochemical reactions in the body that are responsible for the uptake and conversion of nutrients to energy and building blocks for the cell as well as elimination of cellular waste. Metabolism can be divided into catabolism, the breakdown of nutrients to smaller subunits, and anabolism, the build-up of vital cellular building blocks such as proteins and nucleic acids (Maarleveld et al., 2013). In 2011 Hanahan and Weinberg updated their previous observations about the Hallmarks of Cancer with four new characteristics shared by all cancers. One of the new proposed features was reprogramming of energy metabolism, which includes phenomena such as the well-known Warburg effect and enhanced glutamine absorption (Hanahan and Weinberg, 2011). The observed alterations in metabolism as part of tumorigenesis makes the systems biology tool metabolic modeling a suitable approach to studying cancer. Systems biology is an interdisciplinary research field combining both experimental and computational approaches to study complex biological systems as a whole, instead of just the constituting parts (Bosley et al., 2017; Mardinoglu et al., 2018). Advances within biotechnology has led to the development of new high throughput analytical techniques producing large amounts of data. These techniques are often focused on analyzing the genome, transcriptome and proteome, hence generating various types of omics data. Interpreting omics data to gain biological insight is an essential part of systems biology and is done using statistical analysis, network generation and mathematical modeling (Nielsen and Hohmann, 2017).

**FIGURE 1:**
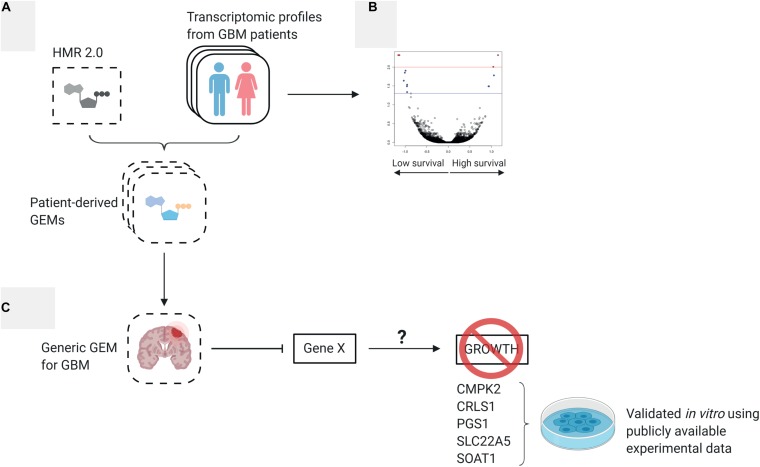
Overview of study workflow **(A)** 139 patient-derived genome scale metabolic models (GEMs) were generated by integrating each patient’s expression data to a generic human metabolic model (Uhlen et al., 2017). The 139 individual GEMs were merged to a generic GBM model. **(B)** The expression profiles used to derive the individual GEMs were analyzed to understand the difference between low survival and high survival patients. **(C)** Essentiality analysis was performed on the generic GBM GEM to find potential therapeutic targets.

In this work, we defined and systematically investigated the transcriptomic differences between high and low survival subgroups of GBM patients from The Cancer Genome Atlas (TCGA) project using systems biology tools. In addition, we employed 139 personalized patient-derived genome-scale metabolic models (GEMs) generated in our previous studies (Uhlen et al., 2017) and performed network dependent analysis, reporter metabolite analysis and gene essentiality analysis to further understand patient heterogeneity from a metabolic perspective. Finally, we integrated the personalized GEMs into a generic GEM for GBM and identified new drug targets that could be used for development of efficient treatment strategies and validated them with publicly available *in vitro* and *in vivo* experimental results. The workflow of this study is depicted in Figure 1.

## Materials and Methods

### RNA-Seq Data

We retrieved RNA-seq data of GBM from TCGA project at the time of the initial release of the Genomic Data Commons (GDC) platform on June 6, 2016 (Grossman et al., 2016). The data set included normalized mRNA expression levels for all protein-coding genes and clinical information (gender, race, disease status, age, and days lived after diagnosis) for 153 patients. To ensure anonymity, each patient is only represented by a TCGA-ID. These IDs are presented in Supplementary Table S5. Mutational status for the genes EGFR, PDGFR alpha, PTEN, and TP53 as well as transcriptomic expression the genes EGFR and PDGFR alpha for each sample was obtained from the cBioPortal (Brennan et al., 2013).

### Differential Gene Expression (DGE) Analysis

We performed DGE analysis between patients with high and low survival. At first, the data set was filtered based on the patient’s status, and only patient’s with vital status “dead” were kept. The survival grouping was done based on the patient’s number of registered living days after diagnosis. The low survival group consisted of the lowest 33% (<231 days) and high survival of the highest 33% (>465 days). The reason for splitting the data as such and not at mean survival was to obtain two groups with a substantial difference in survival, to be able to analyze possible transcriptomic differences due to differing survival times. The expression of all protein-coding genes was compared and results with a *p*value < 0.05 were considered significant. The analysis was done using the R package DESeq2 (Love et al., 2014) in R (R Core Team, 2017). The differentially expressed genes were separated into genes upregulated in the low survival group (log2 fold change < 0) and genes downregulated in the low survival group (log2 fold change > 0). Each list of genes were uploaded to the Database for Annotation, Visualization and Integrated Discovery (DAVID) (Huang et al., 2009) for functional enrichment analysis.

### Co-expression Networks

In order to create co-expression networks, we generated a similarity matrix by pairwise comparison of all genes in the initial data set and calculated the Pearson correlation coefficient for each gene pair. A cutoff for which genes to include in the correlation network was set to 0.99, meaning that only the top 1% correlated gene pairs were kept. The network was constructed from the similarity matrix using the R package igraph (Csardi and Nepusz, 2006). The same package was used for clustering highly co-expressed genes by applying the clustering algorithm Walktrap. Walktrap is a random walk-based method for computing communities in large networks (Pons and Latapy, 2006). Clusters containing a minimum of 5 genes with a correlation coefficient above 0.5 were kept. The co-expression network was visualized using the open source software platform Cytoscape, version 3.6.1 (Shannon et al., 2003).

### Genome-Scale Metabolic Modeling

We downloaded the 139 patient-derived genome-scale metabolic models, each specific to one individual patient (Uhlen et al., 2017) from the BioModels Database (Chelliah et al., 2015) on January 29, 2018. The models were reconstructed using the same set of RNA-seq data from TCGA as used for the DGE analysis above. The reconstruction procedure is described in Uhlen et al. (2017). These 139 models were merged to generate a generic GBM model. For import and all subsequent work with the models the Reconstruction, Analysis and Visualization of Metabolic Networks (RAVEN) Toolbox (Agren et al., 2013) was used. RAVEN runs within MATLAB (version 9.3). Additional installation requirements were MOSEK (version 8.1.0.37, https://www.mosek.com/downloads/) and libSBML (http://sourceforge.net/projects/sbml/files/libsbml/5.4.0/stable/).

To explore the similarity between the 139 models, the Hamming distance between each pair of models was calculated based on the number of differing reactions (reactions only present in one of the models). All distances were collected in a distance matrix (139 × 139) and visualized as a heatmap using the R-package gplots. Hierarchical clustering was performed on the distance matrix and the models were divided into two clusters based on the top split in the dendrogram. Differential expression analysis between the two clusters was performed as described above. The significantly differentially expressed genes (*p* < 0.05) were divided into those upregulated in cluster 1 (log2FC > 0) and those upregulated in cluster 2 (log2FC < 0). The lists of genes were uploaded to the Database for Annotation, Visualization and Integrated Discovery (DAVID) (Huang et al., 2009) for functional enrichment analysis.

### Essentiality Analysis

In silico gene knockout, essentiality analysis (EA), was performed on the chosen models (individual and merged GBM GEMs). Before EA, the model was extended using logic transformation of model (LTM) to ensure that each reaction in the model was only associated to one gene (Zhang et al., 2015). Thereafter, EA was performed using the FastGeneSL command (Zhang et al., 2015) with maximization of biomass (“growth”) as objective function. After a list of essential genes was received, it was investigated whether the knockout of these genes affected the normal brain GEM, obtained from the Human Metabolic Atlas (Pornputtapong et al., 2015). As growth and proliferation is not necessarily the objective of a healthy brain cell another strategy was used to determine the effect on the normal brain. The reactions coupled to the essential genes were iteratively removed from the normal brain GEM and for each removal it was investigated if the cell was able to carry out 77 pre-defined metabolic tasks (Supplementary Table S1; Agren et al., 2014). These tasks include functions that a working cell should be able to perform, such as protein and nucleotide synthesis. If the cell failed to carry out any of the tasks after removal of a gene, this knockout was considered toxic for the normal brain cell in addition to the GBM cell.

### Reporter Metabolite and Subnetwork Analysis

A reporter metabolite analysis (Patil and Nielsen, 2005) was performed based on the results of DGE analysis between the low and high survival GBM patients. In brief, this method is used to identify significantly affected metabolites based on the significance of changes in genes and topology of GEMs. The DGE results were obtained as described above, and the generic GBM model generated in house was used as input for reporter metabolite analysis. The reporter subnetwork was also retrieved using the previously published method (Patil and Nielsen, 2005) with removal of 20 currency metabolites which connected to too many reactions. The deleted currency metabolites are “H2O”, “CO2”, “O2”, “H+”, “HCO3−”, “Na+”, “CoA”, “Pi”, “PPi”, “AMP”, “ADP”, “ATP”, “NAD+”, “NADH”, “NADP+”, “NADPH”, “PAP”, “PAPS”, “FAD,” and “FADH2.”

### Gene Knockout Validation

The Avana gene-knockout effects data set was retrieved from the Supplementary Material of Meyers et al. (2017). The data set initially contained gene-knockout effects scores estimated using the algorithm CERES for 342 cancer cell lines. The gene-knockout effects were obtained by screening with the Avana sgRNA library. The data set was filtered to only keep the 31 glioma cell lines. The median gene dependency score for each of the 5 target genes CMPK2, CRLS1, PGS1, SLC22A5, and SOAT1 was calculated and whether these deviated from the average median gene dependency score for all genes was investigated using Student’s *t*-test.

### Code

The scripts for analysis of the RNAseq data and the essentiality analysis are provided at our dedicated GitHub-repository through the link https://github.com/idalarsson/GEM-for- GBM.git.

## Results

### Differential Gene Expression and Enrichment Analysis

The data set, in its unfiltered format, consisted of expression and clinical data from 153 GBM patients. The mutation frequency and transcriptomic expression for a selection of often altered genes in GBM (TP53, PTEN, EGFR, and PDGFR alpha) for this data set can be seen in Supplementary Figure S2. The percentage of samples affected by mutations in these genes are in accordance with numbers reported for larger GBM cohorts (Brennan et al., 2013). To investigate if there are any significant differences in the gene expression between GBM patients with high and low survival, the data set was divided based on survival time after GBM diagnosis. Stratification generated two groups, low (<231 days) and high (>465 days) survival, holding 40 and 41 patients, respectively. The DGE analysis indicated that 1981 genes were differentially expressed between the two groups. Out of these, 1154 were upregulated and 827 were downregulated in the group with low survival compared to the group with high survival.

**FIGURE 2:**
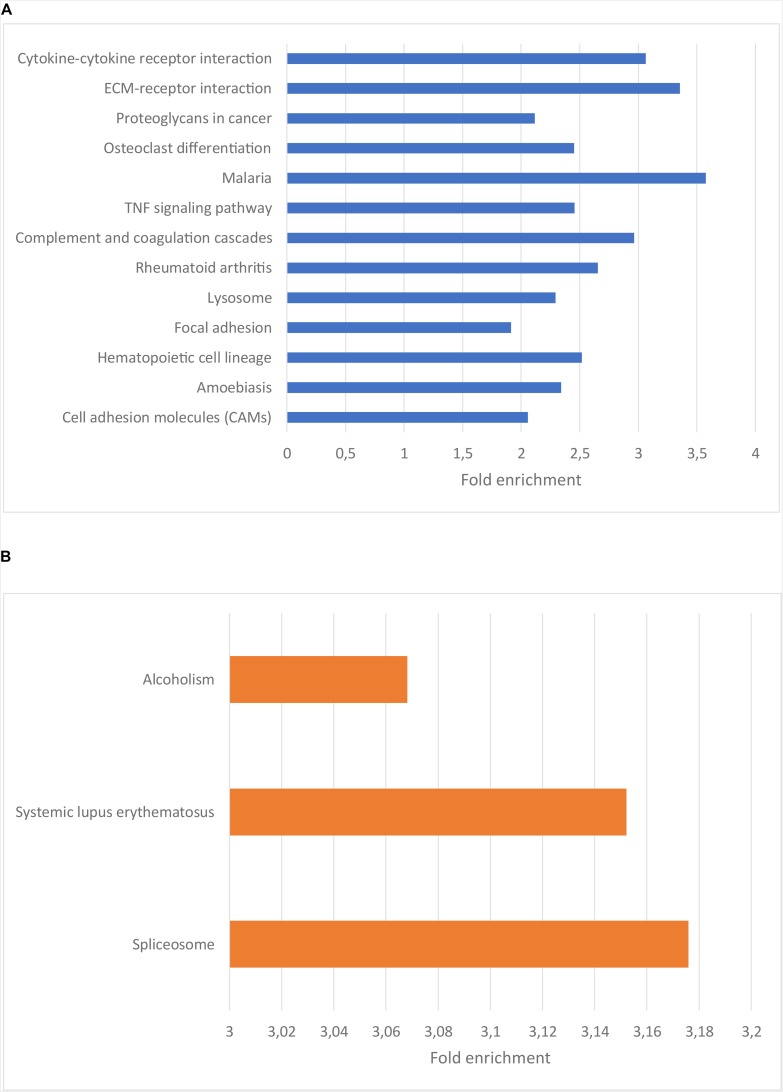
**(A)** Bar plot showing the enriched KEGG pathways for the genes upregulated in the low surival group. The x-axis indicate the fold enrichment for each pathway term. Ordering is based on significance, with the top terms having the highest significance. **(B)** Bar plot showing the enriched KEGG pathways for the genes downregulated in the low surival group. The x-axis indicate the fold enrichment for each pathway term. Ordering is based on significance, with the top terms having the highest significance.

**FIGURE 3:**
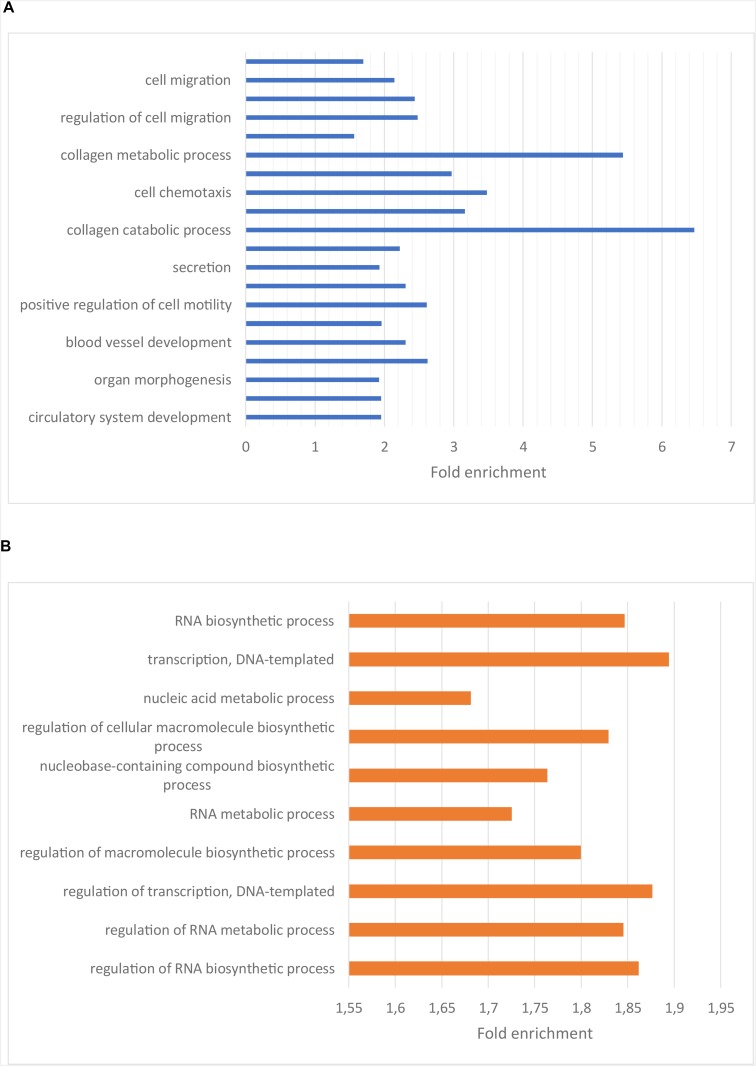
**(A)** Bar plot showing the enriched GO BP terms for the genes upregulated in the low surival group. The x-axis indicate the fold enrichment for each GO BP term. Ordering is based on significance, with the top terms having the highest significance. **(B)** Bar plot showing enriched GO BP terms for the genes downregulated in the low survival group. The x-axis indicate the fold enrichment for each GO BP term. Ordering is based on significance, with the top terms having the highest significance.

To understand the functional importance of these genes, an enrichment analysis was performed using KEGG pathways and Gene Ontology (GO) biological process (BP) terms. The significantly enriched KEGG pathways and a selection of the most significantly enriched GO BP terms are shown in Figures 2, 3, respectively. The result of the enrichment analysis for the upregulated genes indicated that at least a subset of them act in the glioma invasion process, mainly in the interaction with the extracellular matrix (ECM) where detachment from it and breakdown of its components are central (Dou et al., 2012; Cuddapah et al., 2014; Kundu and Forsberg-Nilsson, 2014). GO BP terms relevant for the conclusion are for instance “cell migration,” “collagen metabolic process” and “positive regulation of cell motility.” In addition, the significantly enriched KEGG pathways were important for the drawn conclusion, including “ECM-receptor interaction,” “cytokine-cytokine receptor interaction,” “proteoglycans in cancer” as well as “focal adhesion.” Glioma cell movement is to a high degree dependent on interaction with the ECM. The leading part of a moving cell attach to the matrix through a process mediated by receptors such as integrins and cadherins and the trailing end detach from the matrix by secreting proteases such as matrix metalloproteinases (MMPs). These MMPs also assist the cell by breaking down the matrix when it is hindering the cell to migrate (Cuddapah et al., 2014). On a gene level, genes central for the invasion process such as BDKRB2, MMP2, and MMP9 were all found to be upregulated in the low survival group. As mentioned earlier, tumor recurrence after surgical resection due to GBM cell migration is a major issue when treating GBM. A more aggressive migration pattern, possibly leading to more rapid tumor recurrence, could therefore be a reasonable explanation for the shorter survival times of the low survival group. Attempts to counteract the invasion process have been tried as therapeutic strategies in clinical trials (Cuddapah et al., 2014) and the result obtained here further motivate these endeavors.

Regarding the genes downregulated in the low survival group, the enriched KEGG terms, e.g., “alcoholism” and “spliceosome” (Figure 2B), weren’t as related to the oncological process as those described above. The enriched GO BP terms for the downregulated genes are to a high degree related to the production and function of RNA (Figure 3B), e.g., “RNA biosynthetic process,” “regulation of transcription,” and “RNA metabolic process.”

**FIGURE 4:**
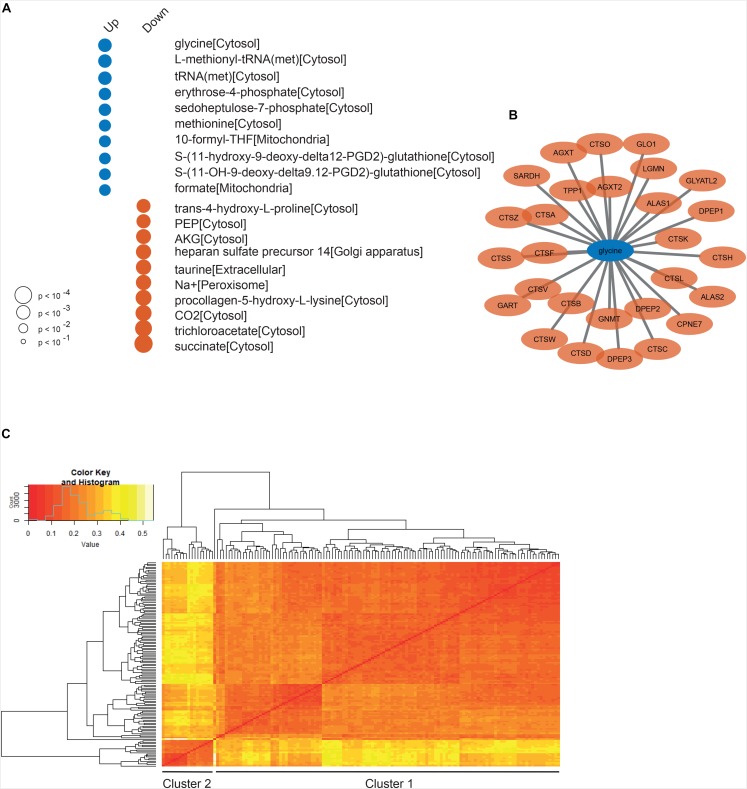
**(A)** The top scoring reporter metabolites associated with up- and downregulated genes in the low survival group. The size of the dot indicate significance (*p*-value) and the color whether the metabolite is associated with up- (blue) or downregulated (red) genes. **(B)** a detailed view of the part of the reporter sub network with genes directly connected to the top-scoring metabolite glycine. **(C)** Heatmap of the distance matrix between all individual GEMs, where each line at the end of the dendrogram corresponds to one individual GEM.

To reveal the altered metabolism, we also performed reporter metabolite analysis using the result of DGE analysis between the low and high survival GBM patients and the generic GEM for GBM (Figure 4A). We found that the top scoring reporter metabolites associated with genes upregulated in the low survival group are involved in the nucleotide biosynthesis and the pentose phosphate pathway (PPP). It is a known fact that tumor cells redirect their carbon metabolism from glycolysis to the PPP in order to increase nucleotide synthesis to enable proliferation (Agnihotri and Zadeh, 2016). Interestingly, we see that glycine is the top scoring reporter metabolite for patients with low survival. An increased uptake of glycine in high-grade gliomas, such as glioblastomas, compared to low-grade gliomas have previously been demonstrated (Hattingen et al., 2009; Chinnaiyan et al., 2012), leading to glycine being suggested as a potential marker of tumor grade. Our results could indicate that there is a further connection between glycine content and survival within the high-grade glioma. Similar observations have been made in a study made by Jain et al., which showed that uptake and catabolism of glycine correlate with the rate of cancer cell proliferation and poor survival of cancer patients across various cancer types. Moreover, we performed reporter subnetwork analysis to find the key genes and metabolites associated with the low survival of the GBM patients. We found that glycine is directly connected to 28 genes in the network (Figure 4B). Enrichment analysis performed on these genes indicate that they are involved in processes such as “protein catabolic process” and “proteolysis.”

### Essentiality Analysis Using GEMs for GBM

To further investigate GBM from a metabolic perspective, we employed 139 patient-derived GEMs from our previous study (Uhlen et al., 2017) and performed network and GEM based analysis. First of all, we calculated the Hamming distance between each pair of models and compiled a distance matrix in order to explore the difference between the individual models. As shown in Figure 4C, it is evident that a cluster of 18 models differs from the rest of the models. Based on the top split in the dendrogram, these 18 models were assigned to one cluster (cluster 2) and the remaining ones to another one (cluster 1). Functional enrichment analysis on differentially expressed genes between the two clusters revealed that genes upregulated in cluster 1 were associated with the cell’s immune response, e.g., the GO BP terms “defense response,” “T cell activation” and “immune system response,” suggesting a link between tumor metabolism and immune response. We also observed that the patients in cluster 2 had a higher mean survival (487 days) than those in cluster 1 (375 days), but the difference was not statistically significant (Student’s *t*-test, *p* = 0.23), which suggests that survival outcome of the patients is not decided by the tumor metabolism alone. However, for the interested we did perform gene essentiality analysis on GEMs from high and low survival patients separately. The result from this analysis can be seen in Supplementary Table S3.

Essentiality analysis to find potential therapeutic targets was performed on a generic GEM for GBM. The generic GEM for GBM was generated by merging 139 patient-derived GEMs, and thus, the essentiality analysis is expected to find therapeutic targets with the potential of being effective in all GBM patients rather than just a subset of them. The essentiality analysis generated a list of 24 genes that when removed from the GBM model each caused the cell to fail fulfilment of its objective function, growth (Supplementary Table S2). To ensure that these identified gene targets are in fact essential in all 139 models, we extended the essentiality analysis and performed it on each of the 139 patient-derived GEMs individually, which resulted in a list of 96 unique genes that are to a minimum essential in one GEM. The 24 essential genes mentioned above were indeed essential for 100% of the patient-derived GEMs (Supplementary Table S2).

Since the goal of this simulation was to find potential therapeutic targets, one important consideration is that a future treatment should be as gentle as possible toward the normal brain tissue. The reactions coupled to the 24 essential genes were therefore iteratively removed from a healthy brain GEM to evaluate their potential toxicity. For each removal it was investigated if the normal brain cell could carry out essential metabolic tasks that were previously defined (Agren et al., 2014), such as protein and nucleotide synthesis. If the cell failed any of the tasks after the *in silico* gene knockout, this gene was considered a toxic target for the normal brain cell in addition to the GBM cell, and thus abrogated as a drug target. Consequently, the 24 genes could be narrowed down to five genes including SOAT1, PGS1, CRLS1, CMPK2 and SLC22A5, which did not affect the essential metabolic tasks in the GEM for the healthy brain. The model was still capable of carrying out 77 pre-defined common biological tasks for a cell even though lacking the reactions coupled to the removed gene.

### Validation and Function of the Found Essential Genes

We used CRISPR derived CERES gene dependency scores (Meyers et al., 2017) as a first step to validate our predictions. The median gene dependency score is −1 and 0 for essential and non-essential genes, respectively. All five genes have a negative median gene dependency score (Supplementary Figure S1), indicating some degree of essentiality for all five genes in the 31 glioma cell lines included. In addition, comparing the five genes against the distribution of all genes, PGS1 and CRLS1 have significantly smaller CERES gene dependency scores (Student’s *t*-test, p_PGS1_ < 2.2 10^–16^ and p_CRLS1_ = 0.01455), which indicates that these two genes are particularly essential for glioma cell lines and together with our model based predictions, suggests they could be promising novel drug targets.

Previous associations between the five genes and GBM were also examined by performing a combined PubMed-search on the five gene names and “glioblastoma.” The only gene that had previously been clearly connected to GBM was SOAT1, whose inhibition suppressed GBM growth in mice (Geng et al., 2016). Briefly, it was demonstrated that SOAT1 contributes to maintained cholesterol homeostasis in the endoplasmic reticulum (ER) during tumorigenesis through formation of lipid droplets. Homeostasis enables the SREBP1-complex located in the ER membrane to dissociate and be transported to the Golgi apparatus. There it is cleaved and the now active N-terminal is transported to the nucleus where it acts as a transcription factor to activate lipogenesis. This in turn promotes tumor growth. When SOAT1 is inhibited, the cholesterol accumulation in the ER prevents SBREP1 to relocate to the Golgi, hence lipogenesis is not activated, and tumor growth is suppressed. This work can be regarded as an *in vitro* validation of the results obtained through the *in silico* simulation in this project. A relevant additional finding here is that the inhibition of SOAT1 does not seem to affect the normal brain cell model.

According to literature, the remaining four of the five genes have not been connected to GBM in previous studies, therefore they could be potential new targets. Among these genes, PGS1 and CRLS1 show most promising according to the gene dependency scoring. PGS1 encodes mitochondrial CDP-diacyl-glycerol-3-phosphate-3-phosphatidyltransferase, which has a function in the biosynthesis of anionic phospholipids, phosphatidylglycerol and cardiolipin and CRLS1 encodes cardiolipin synthase, which catalyzes the formation of cardiolipin. In addition, CMPK2 encodes mitochondrial UMP-CMP kinase 2, which may have a function in the synthesis of dUTP and dCTP and SLC22A5 encodes solute carrier family 22 member 5 that is involved in the cell’s uptake of carnitine, which in turn is responsible for transporting fatty acids across the mitochondrial membrane (UniProt Consortium, 2018).

Evidently, three of the remaining four essential genes have a clear role in lipid metabolism, e.g., cardiolipin synthesis and carnitine transport. Elevated lipid levels and reprogramming of lipid metabolism have previously been reported for GBM and findings related to lipid metabolism are therefore to be expected (Geng and Guo, 2017). One of the three genes is SLC22A5, a membrane protein responsible for the cellular uptake of carnitine (UniProt Consortium, 2018). The important role of carnitine in the fatty acid oxidation process could be hypothesized as the reason for SLC22A5 being essential for the GBM cell. Recent studies performed on patient-derived GBM cells have shown that the cells are more dependent on fatty acid oxidation (FAO) as glycolytic pathway than previously thought, and that inhibition of FAO in GBM-induced mice prolonged their survival (Lin et al., 2017). In the study, inhibition of FAO was done by suppressing the enzyme carnitine palmitoyl transferase 1 (CPT1) using the already existing drug Etomoxir. As the name implies, CPT1 is dependent on carnitine for its function (Prip-Buus et al., 2001). By inhibiting SLC22A5, the cell’s supply of carnitine would decrease, which in theory could work as an indirect inhibition of CPT1 and the FAO process. Currently, there are anti-cancer drugs on the market with the purpose of inhibiting SLC22A5. It has been noted that inhibiting SLC22A5 could lead to secondary carnitine deficiency (Longo et al., 2016), a disease state that could give rise to problems such as muscle weakness for affected patients. The potential side effects of inhibiting SLC22A5 to treat glioblastoma must of course be taken into consideration and evaluated.

The fifth gene, CMPK2, appears to be an outlier in terms of function. It is reported to be involved in the synthesis of the nucleotides dUTP and dCTP in the mitochondria (UniProt Consortium, 2018). CMPK2 is a key gene in DNA synthesis, and is therefore essential for cell growth which is elevated in tumor cells but not in normal brain cells.

**FIGURE 5:**
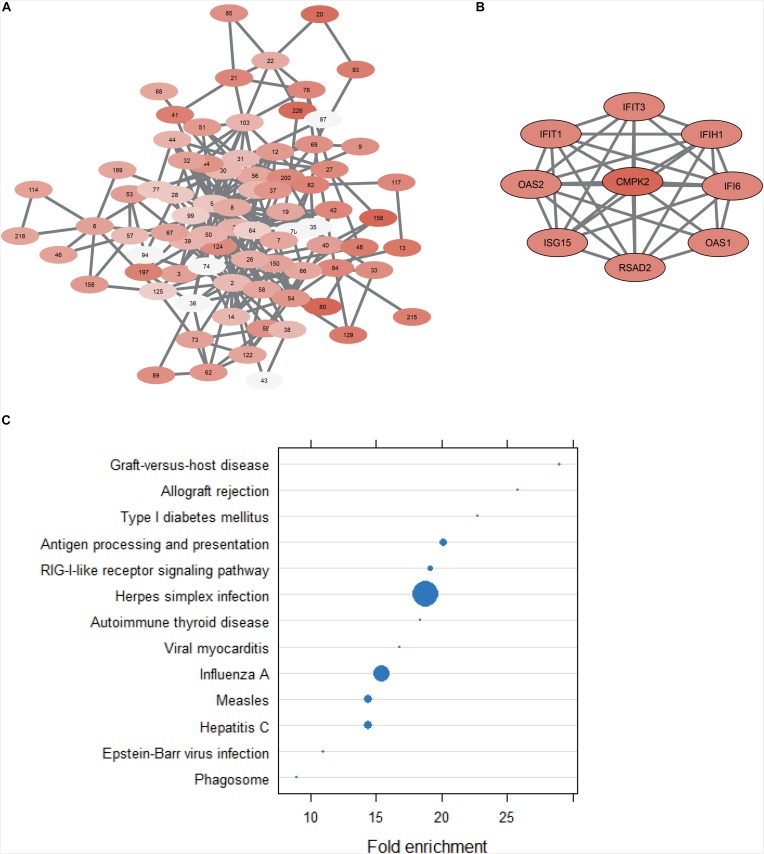
**(A)** Co-expression network derived from the expression profiles of all 139 GBM patients. Nodes indicate clusters of co-expressed genes while the edges indicate significant links between clusters. **(B)** Part of node 42 showing the gene CMPK2 and its eight most correlated genes. **(C)** Dot plot of the results from functional enrichment analysis with KEGG database of all 90 genes in node 42. All included terms are significant (FDR < 0.25), but the size of the dot is inversely proportional to the adjusted *p*-value (the larger the dot, the smaller the *p*-value).

To further investigate the role of CMPK2 in the context of GBM, the results of the co-expression analysis was consulted (Supplementary Table S4). The generated co-expression network can be seen in Figure 5A. Each node represents a cluster of highly correlated genes. The findings suggest that CMPK2 is connected to the cellular response to a viral infection. Firstly, its eight most correlated genes in terms of expression (correlation coefficient above 0.9) (Figure 5B) have all, in various ways, antiviral activity with the majority being interferon-induced. Secondly, when investigating the co-expression cluster that CMPK2 belongs to (cluster 42) and performing enrichment analysis on the 90 included genes, both obtained GO BP terms and enriched KEGG pathways are exclusively related to virus infection and cellular defense response (Figure 5C). The presence of human cytomegalovirus (HCMV) in brain tumor cells and its potential role in the development and progression of GBM has been, and remains, a highly debated and controversial topic in the field of GBM research. Many research groups have independently shown that HCMV is present in GBM tumor cells but not in the surrounding healthy brain tissue (Cobbs, 2013), while other publications contradict these findings and claim that their experiments detect no presence of HCMV in GBM tumor cells (Johnson et al., 2017). Further work must be done on the connection between viral infections and the altered essentiality of CMPK2. Based on the collected findings of this project, it could potentially be a promising target for developing therapies that explore the possible viral cause of GBM.

## Discussion

We employed a systems biology approach to reveal the underlying molecular mechanism involved in the progression of aggressive brain cancer. To investigate if there are differences in gene expression between patients with high and low survival, the data set consisting of data from 153 patients were divided based on survival time after GBM diagnosis. We first focused on analysis of gene expression data between low and high survival GBM patients and identified the enriched KEGG pathways and GO BP terms. We found that genes upregulated in the patients with low survival are related to cell migration and the glioma invasion process.

One limitation regarding the stratification of GBM patients is that not all information on the stage of the disease at time of diagnosis are available. Patients with advanced GBM at time of diagnosis could bias the selection, or vice versa. However, the current stratification was the possible one based on available clinical information.

Next, we generated a generic GEM for GBM patients and employed it in the analysis of DGE between low and high survival patients. By applying the reporter metabolites and subnetwork algorithms, we found key metabolites associated with low survival of the GBM patients. We found that glycine as well as metabolites involved in nucleotide biosynthesis and the pentose phosphate pathway (PPP) were key metabolites.

To identify potential therapeutic targets that can be used in the development of new drugs, we performed gene essentiality analysis using the generic GEM for GBM patients. We identified five genes that were essential for the growth of GBM and at the same time non-toxic to remove from healthy brain tissue. These genes were CMPK2, CRLS1, PGS1, SLC22A5, and SOAT1. By investigating both publicly available gene dependency data sets and literature, we found *in vitro* or *in vivo* evidence of the essentiality of 4 out of the 5 found genes in GBM.

Moving further, the next step would be to perform *in vitro* experiments to validate the suggested therapeutic targets’ negative effect on tumor growth. As was done for SOAT1 (Geng et al., 2016), an experimental setup that could elucidate the mechanism behind suppressed tumor growth is desirable, to test the hypotheses proposed in this paper.

GBM is a very aggressive cancer with poor prognosis for the affected patients. The current line of treatment is inadequate, only giving the patients an average survival time of 15 months (Ostrom et al., 2013). In this project, the aim was to use a systems biology approach to investigate the disease from a systemic point of view. The findings span various aspects of GBM pathogenesis, from glioma invasion to a viral source of the disease, which emphasize the strength of applying a holistic approach to cancer research in general and drug target identification in particular. The work presented in this paper add to the current knowledge of GBM and could contribute to new findings leading to better treatment for the patients.

## Data Availability Statement

Publicly available datasets were analyzed in this study. This data can be found here: https://portal.gdc.cancer.gov/projects/TCGA-GBM, https://www.ebi.ac.uk/biomodels-main/pdgsmm?disease=Glioblastoma%20Multiforme, https://www.metabolicatlas.org/gems/repository, doi: 10.1038/ng.3984, https://github.com/idalarsson/GEM-for-GBM.git.

## Author Contributions

CZ, AM, and MU designed the study and assisted in the analysis. IL performed most of the analysis. IL wrote the manuscript. All authors were involved in editing the manuscript.

## Conflict of Interest

The authors declare that the research was conducted in the absence of any commercial or financial relationships that could be construed as a potential conflict of interest.

## Supplementary Material

FIGURE S1 **(A)** The median gene dependency score for 5 essential genes in 31 glioma cell lines. **(B)** Histogram of the median gene dependency score for all gene in he 31 glioma cell lines.

FIGURE S2 Mutational frequency and transcriptomic expression for selected genes. Each column corresponds to one patient. Top row showing the spread of overall survival for the patients included in this study. Row 2–5 shows the mutational status for the genes EGFR, PDGFRA, PTEN, and TP53 in the GBM patients. The meaning of the colors are explained in the section “Genetic Alteration.” Row 6, 7 shows transcriptomic expression for the genes EGFR and PDGFRA, color key in the section “Expression Heatmap.”

TABLE S1 The 77 pre-defined metabolic tasks that a healthy cell should be able to carry out.

TABLE S2 Results from the essentiality analysis on the 139 individual GEMS.

TABLE S3 Gene essentiality analysis for high and low survival patients, respectively. We respectively, applied gene essentiality analysis to the patient derived GEMs of high and low survival patient groups used for the DGE analysis. The majority of the essential genes were shared between the two subgroups, except for 5 genes that were essential in high survival GEMs but not in low survival GEMs, and 7 genes essential in low survival GEMs but not in high survival GEMs. The genes and their metabolic functions are summarized below.

TABLE S4 Specifications of the co-expression analysis. Table 1: Genes included in each node cluster. Table 2: Node file used as input to Cytoscape. Table 3: Edge file used as input to Cytoscape.

TABLE S5 TCGA-ID for the analyzed patients.
