# Supplementary material for: Genome-Scale Metabolic Modeling of Glioblastoma Reveals Promising Targets for Drug Development
Source: Front Genet. 2020 Apr 17;11:381. doi: 10.3389/fgene.2020.00381 (PMC7181968; doi:10.3389/fgene.2020.00381)
Supplement: FIGURE S1 — (A) The median gene dependency score for 5 essential genes in 31 glioma cell lines. (B) Histogram of the median gene dependency score for all gene in he 31 glioma cell lines. [file Data_Sheet_1.ZIP › Data sheet 1_revised_25mars/Figure S2.pdf]

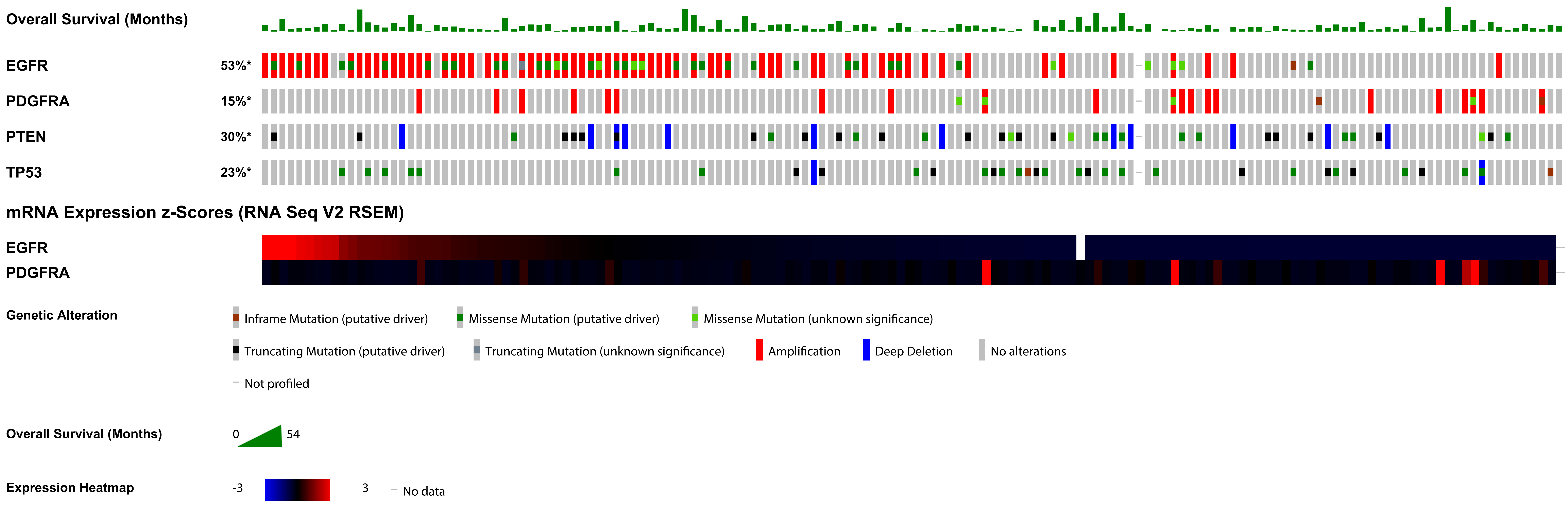

Figure S2: Mutational frequency and transcriptomic expression for selected genes. Each column corresponds to one patient. Top row showing the spread of overall survival for the patients included in this study. Row 2-5 shows the mutational status for the genes EGFR, PDGFRA, PTEN and TP53 in the GBM patients. The meaning of the colors are explained in the section "Genetic Alteration". Row 6-7 shows transcriptomic expression for the genes EGFR and PDGFRA, color key in the section "Expression Heatmap".
