# Supplementary material for: Genome-Scale Metabolic Modeling of Glioblastoma Reveals Promising Targets for Drug Development
Source: Front Genet. 2020 Apr 17;11:381. doi: 10.3389/fgene.2020.00381 (PMC7181968; doi:10.3389/fgene.2020.00381)
Supplement: FIGURE S1 — (A) The median gene dependency score for 5 essential genes in 31 glioma cell lines. (B) Histogram of the median gene dependency score for all gene in he 31 glioma cell lines. [file Data_Sheet_1.ZIP › Data sheet 1_revised_25mars/Supplementary Table S3.docx]

***Gene essentiality analysis for high and low survival patients respectively***

We respectively applied gene essentiality analysis to the patient derived GEMs of high and low survival patient groups used for the DGE analysis. The majority of the essential genes were shared between the two subgroups, except for 5 genes that were essential in high survival GEMs but not in low survival GEMs, and 7 genes essential in low survival GEMs but not in high survival GEMs. The genes and their metabolic functions are summarized below.

| **Genes essential in high survival GEMs** | **Function** |
| --- | --- |
| SLC25A1 | Citrate transporter |
| GPI | Mediates GPI anchoring in the endoplasmic reticulum |
| TPI1 | Catalyses the interconversion between dihydroxyacetone phosphate (DHAP) and D-glyceraldehyde-3-phosphate (G3P) in glycolysis and gluconeogenesis |
| UMPS | Uridine 5'-monophosphate synthase |
| ADA | Adenosine deaminase. Plays an important role in purine metabolism and in adenosine homeostasis |

| **Genes essential in low survival GEMs** | **Function** |
| --- | --- |
| SPHK2 | Catalyzes the phosphorylation of sphingosine to form sphingosine-1-phosphate (SPP) |
| SULT2B1 | Sulfotransferase 2B1. Preferentially sulfonates cholesterol |
| ACOT8 | Acyl-coenzyme A thioesterase 8. Acyl-coenzyme A (acyl-CoA) thioesterases are a group of enzymes that catalyze the hydrolysis of acyl-CoAs to the free fatty acid and coenzyme A (CoASH) |
| STS | Catalyzes the conversion of sulfated steroid precursors, such as dehydroepiandrosterone sulfate (DHEA-S) and estrone sulfate to the free steroid |
| GPAT4 | Converts glycerol-3-phosphate to 1-acyl-sn-glycerol-3-phosphate (lysophosphatidic acid or LPA) by incorporating an acyl moiety at the sn-1 position of the glycerol backbone. |
| PCYT1A | Controls phosphatidylcholine synthesis. |
| UGP2 | Plays a central role as a glucosyl donor in cellular metabolic pathways. |
