# Supplementary figures and images for: Genome-Scale Metabolic Modeling of Glioblastoma Reveals Promising Targets for Drug Development
Source: Front Genet. 2020 Apr 17;11:381. doi: 10.3389/fgene.2020.00381 (PMC7181968; doi:10.3389/fgene.2020.00381)

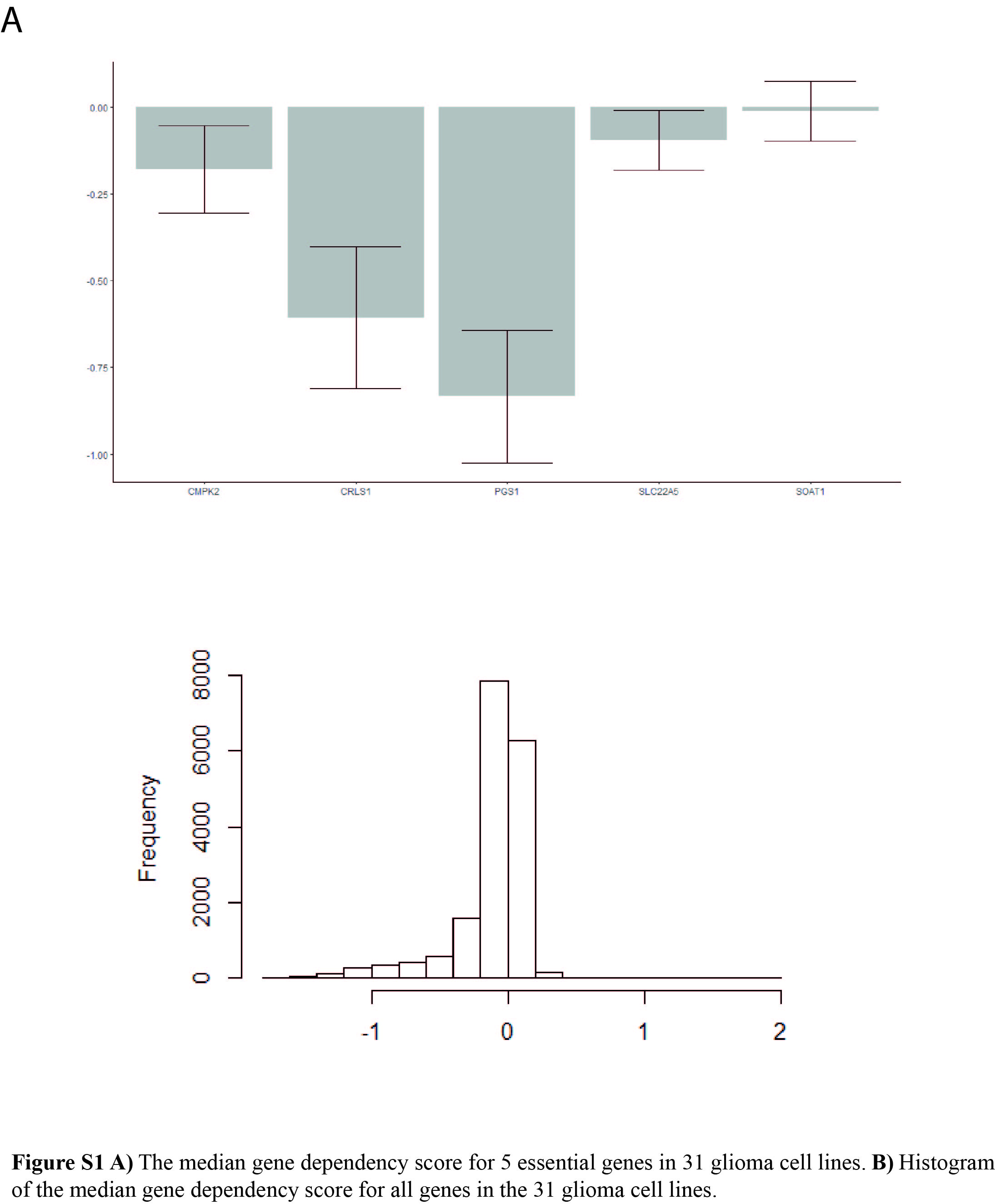

Supplement: FIGURE S1 — (A) The median gene dependency score for 5 essential genes in 31 glioma cell lines. (B) Histogram of the median gene dependency score for all gene in he 31 glioma cell lines. [file Data_Sheet_1.ZIP › Data sheet 1_revised_25mars/Figure S1.jpg]

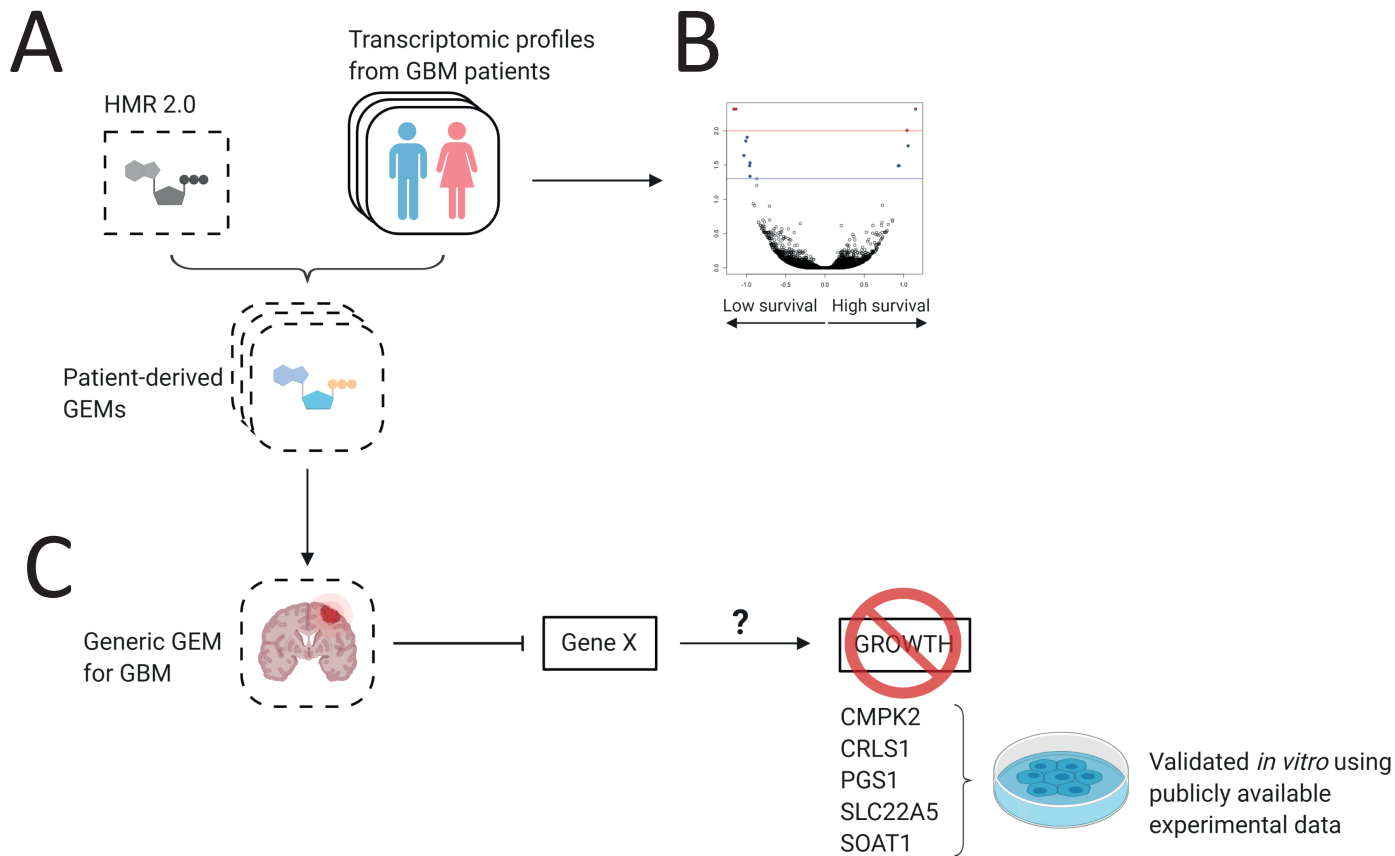

Supplement: Supplementary file 2 [file Image_1.pdf]

A

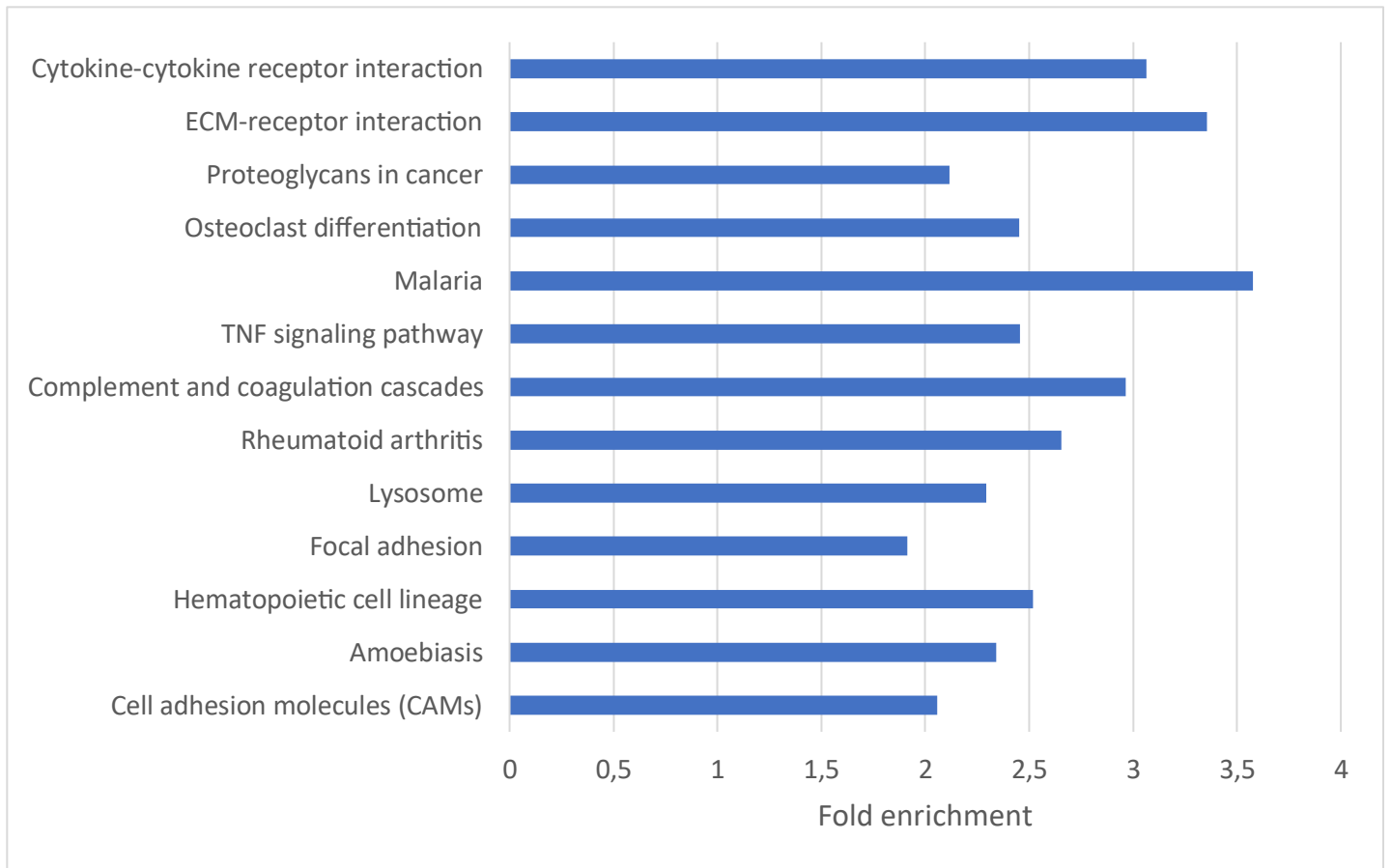

B

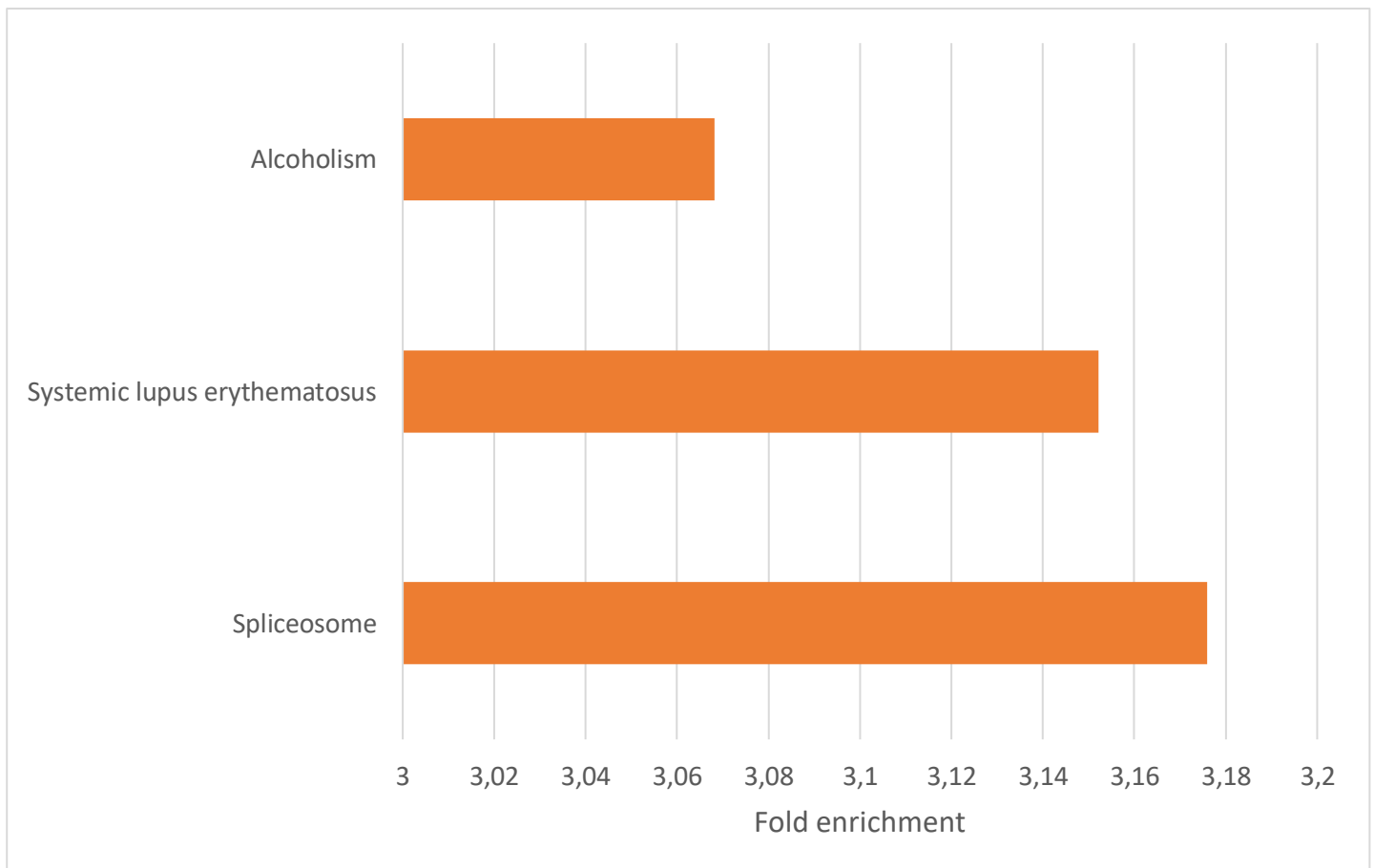

Supplement: Supplementary file 3 [file Image_2.pdf]

A

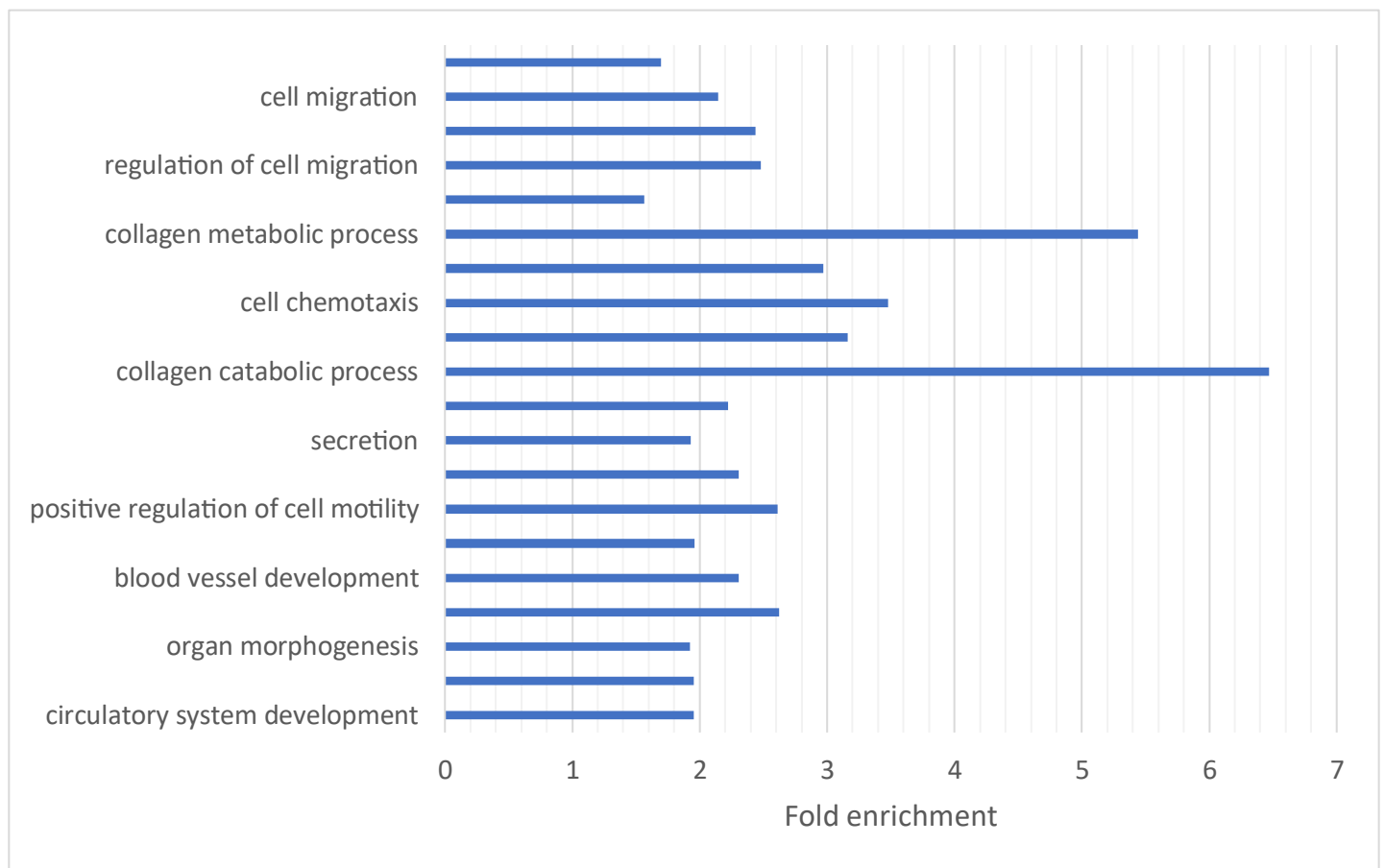

B

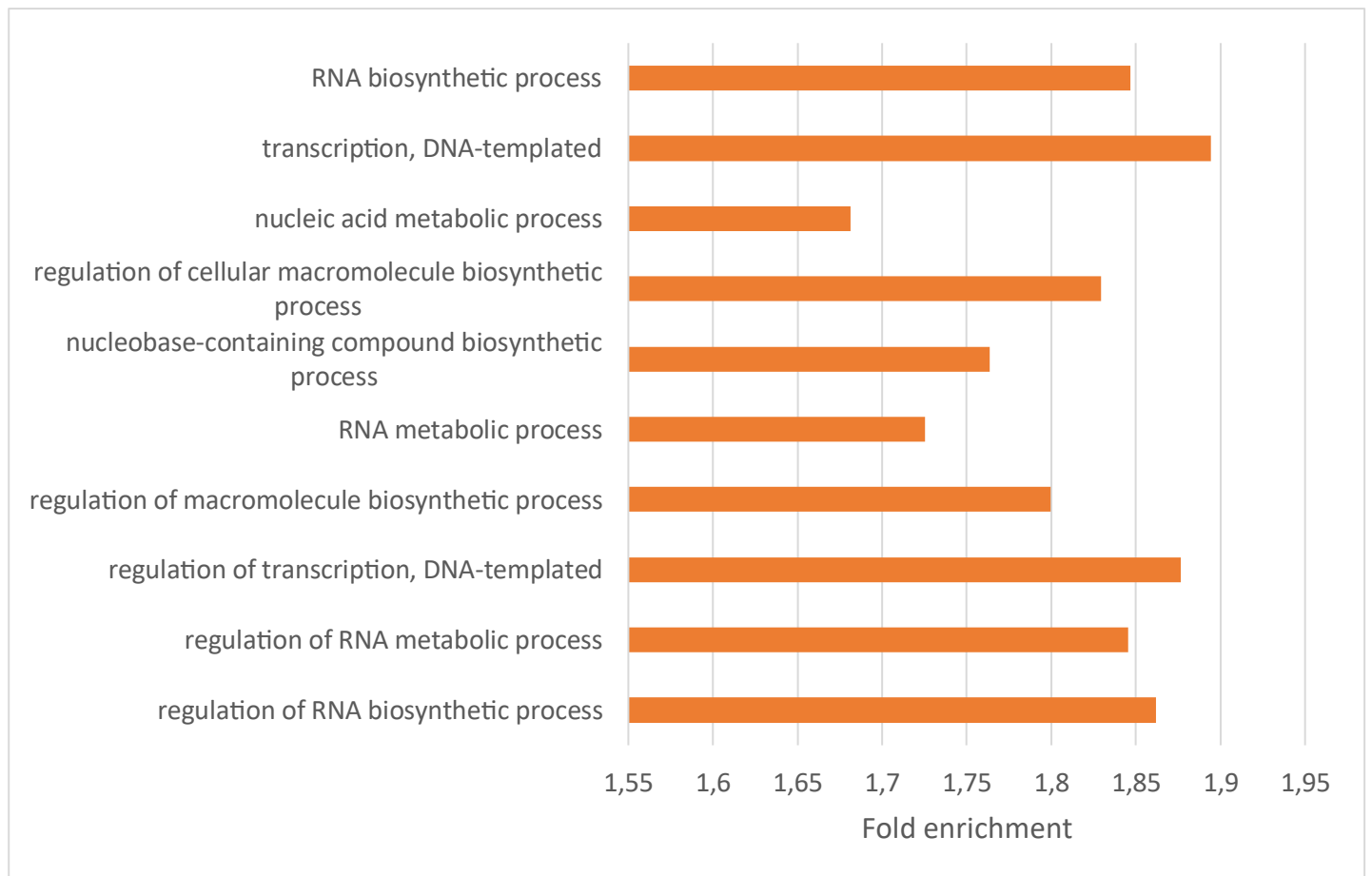

Supplement: Supplementary file 4 [file Image_3.pdf]

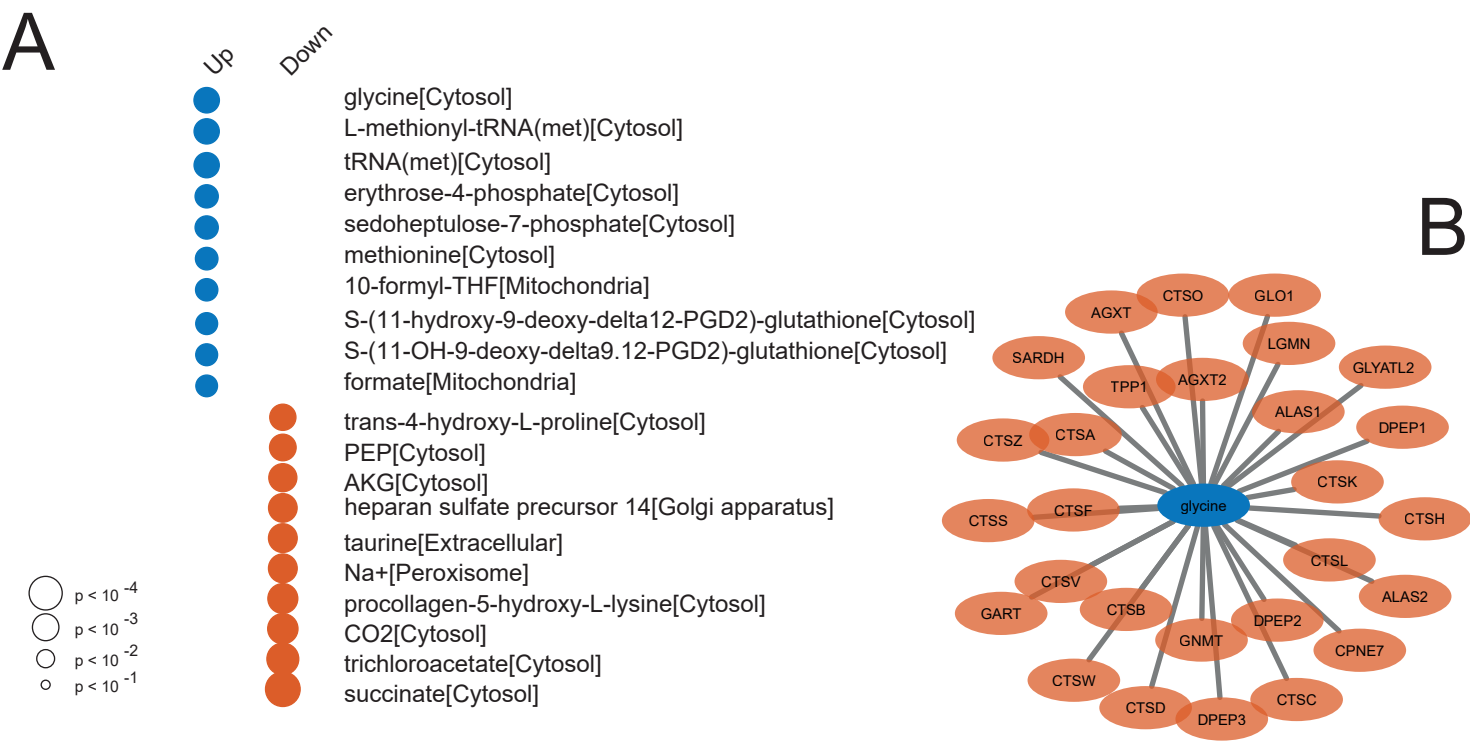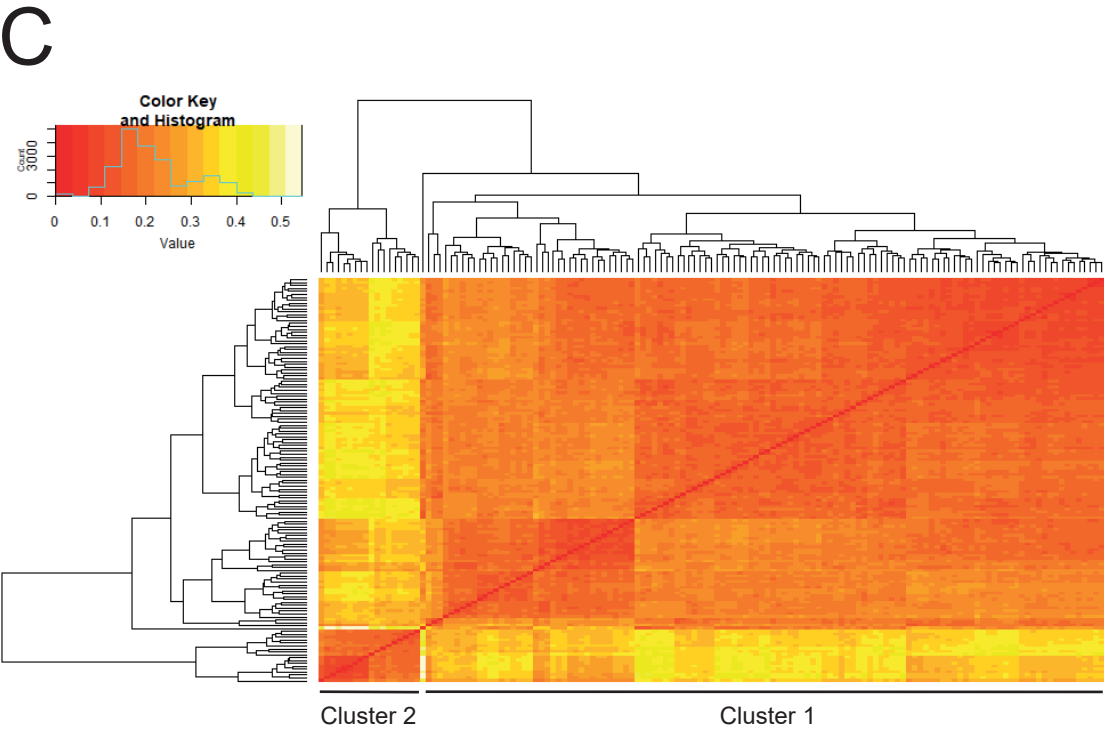

Supplement: Supplementary file 5 [file Image_4.pdf]

A

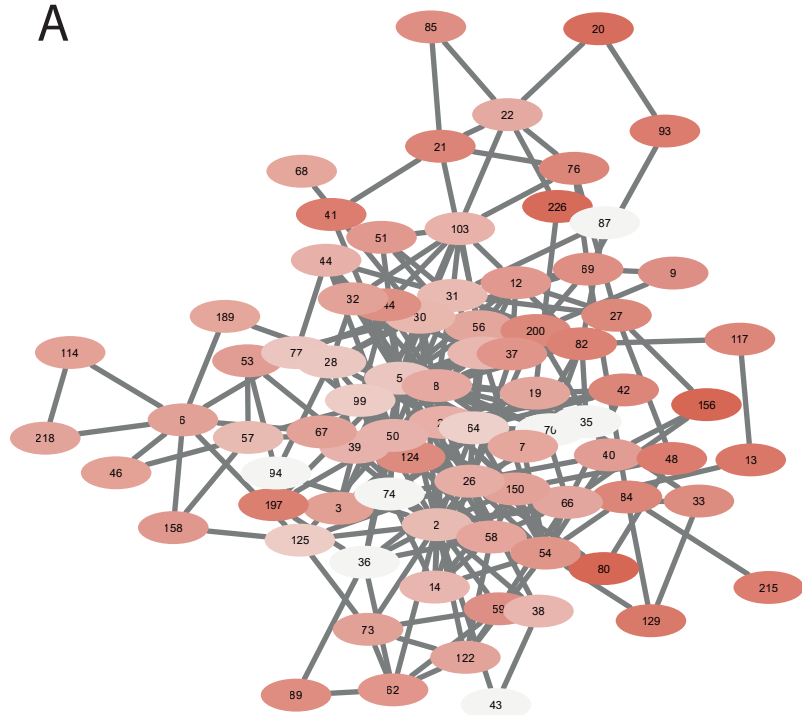

B

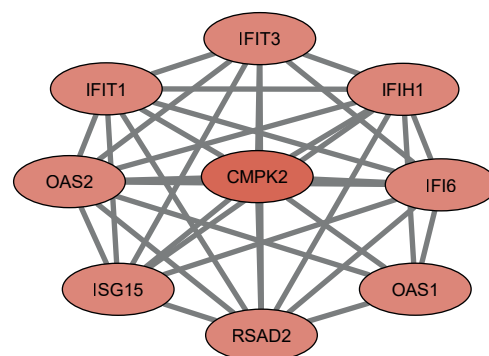

C

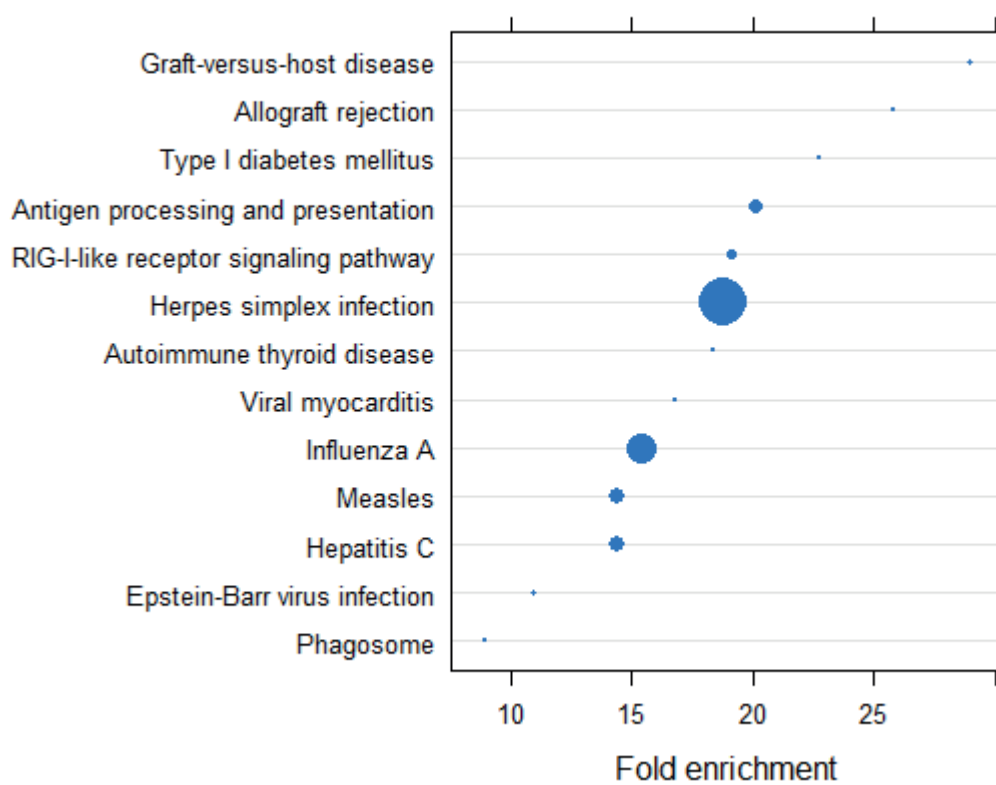

Supplement: Supplementary file 6 [file Image_5.pdf]
